# Relating Gut Microbiome and Its Modulating Factors to Immunotherapy in Solid Tumors: A Systematic Review

**DOI:** 10.3389/fonc.2021.642110

**Published:** 2021-03-18

**Authors:** Chengliang Huang, Meizhang Li, Ben Liu, Huanbo Zhu, Qun Dai, Xianming Fan, Kathan Mehta, Chao Huang, Prakash Neupane, Fen Wang, Weijing Sun, Shahid Umar, Cuncong Zhong, Jun Zhang

**Affiliations:** ^1^Department of Respiratory and Critical Care Medicine, The Affiliated Hospital of Southwest Medical University, Luzhou, China; ^2^Division of Medical Oncology, Department of Internal Medicine, University of Kansas Cancer Center, University of Kansas Medical Center, Westwood, KS, United States; ^3^Department of Pathology and Laboratory Medicine, University of Kansas Medical Center, Kansas City, KS, United States; ^4^Department of Electrical Engineering and Computer Science, University of Kansas, Lawrence, KS, United States; ^5^Department of Gastrointestinal Surgery, The Second Affiliated Hospital & Yuying Children's Hospital of Wenzhou Medical University, Wenzhou, China; ^6^Department of Radiation Oncology, University of Kansas Cancer Center, University of Kansas Medical Center, Kansas City, KS, United States; ^7^Department of Surgery, University of Kansas Cancer Center, University of Kansas Medical Center, Kansas City, KS, United States; ^8^Department of Cancer Biology, University of Kansas Cancer Center, University of Kansas Medical Center, Kansas City, KS, United States

**Keywords:** gut microbiome, modulating factors, immunotherapy, solid tumors, diet, microbiota, antibiotics, cancer immunotherapy

## Abstract

**Background:** Gut microbiome is proved to affect the activity of immunotherapy in certain tumors. However, little is known if there is universal impact on both the treatment response and adverse effects (AEs) of immune checkpoint inhibitors (ICIs) across multiple solid tumors, and whether such impact can be modulated by common gut microbiome modifiers, such as antibiotics and diet.

**Methods:** A systematic search in PubMed followed by stringent manual review were performed to identify clinical cohort studies that evaluated the relevance of gut microbiome to ICIs (response and/or AEs, 12 studies), or association of antibiotics with ICIs (17 studies), or impact of diet on gut microbiome (16 studies). Only original studies published in English before April 1st, 2020 were used. Qualified studies identified in the reference were also included.

**Results:** At the phylum level, patients who had enriched abundance in *Firmicutes* and *Verrucomicrobia* almost universally had better response from ICIs, whereas those who were enriched in *Proteobacteria* universally presented with unfavorable outcome. Mixed correlations were observed for *Bacteroidetes* in relating to treatment response. Regarding the AEs, *Firmicutes* correlated to higher incidence whereas *Bacteroidetes* were clearly associated with less occurrence. Interestingly, across various solid tumors, majority of the studies suggested a negative association of antibiotic use with clinical response from ICIs, especially within 1-2 month prior to the initiation of ICIs. Finally, we observed a significant correlation of plant-based diet in relating to the enrichment of “ICI-favoring” gut microbiome (*P* = 0.0476).

**Conclusions:** Gut microbiome may serve as a novel modifiable biomarker for both the treatment response and AEs of ICIs across various solid tumors. Further study is needed to understand the underlying mechanism, minimize the negative impact of antibiotics on ICIs, and gain insight regarding the role of diet so that this important lifestyle factor can be harnessed to improve the therapeutic outcomes of cancer immunotherapy partly through its impact on gut microbiome.

## Introduction

Immunotherapy such as using immune checkpoint inhibitors (ICIs) targeting PD-1/L1 and CTLA-4 has revolutionized our management of various cancer types including lung cancer (1, 2). However, only a subset of patients derive the benefit, which can be further limited by AEs especially immune-related AEs (irAEs) (3). The gut microbiome, due to its close interaction with immune system, has gained increasing attention for its potential role in cancer immunotherapy (4, 5). This is supported by several preclinical models (6, 7), as well as correlative studies at the human level including ours (8). However, several key questions remain to be addressed: (1) whether there is shared feature of gut microbiome that links to ICI response and AEs across various solid tumors; (2) whether antibiotics can affect cancer immunotherapy. This is important considering there are controversial results (6, 9–11), and antibiotic is such an inevitable gut microbiome modifier in the clinical setting; (3) whether diet, as one of the most important lifestyle factors, will have impact on cancer immunotherapy. We aim to investigate existing evidence that could help address these questions at the human level using a systematic review.

## Methods

This systematic review focused on bacterial gut microbiome. Different search keywords and their combination were used to extract relevant clinical studies from PubMed to address each proposed question. This was followed by a stringent manual selection to include only relevant studies, including those identified in the references. To explore the relationship between gut microbiome and clinical outcomes from ICIs, we used search keywords “gut microbiome” AND “cancer” AND “immunotherapy.” To determine the impact of antibiotics on ICIs, we used keywords “antibiotics” AND “immunotherapy” AND “microbiome” AND “cancer.” To investigate the impact of diet on gut microbiome, we used “diet” AND “gut microbiome” AND “healthy adult” with series of keyword refinements as detailed below. Only original clinical studies in human subjects written in English, with the publishing date before Apr 1st, 2020 (Supplementary Table 1) were included in this review to draw meaningful conclusion at the human level. Various key information such as gut microbiome data, clinical outcome (e.g., therapeutic response and AEs), timing and duration of antibiotic use, and diet were extracted and used to address separate but coherent questions with details below. Descriptive statistics was used to summarize the study findings. Fisher's test was used for the comparisons between 2 groups, and a *P* < 0.05 was considered statistically significant.

## Results

### Common Features in Gut Microbiome Correlate With the Treatment Response and AEs of ICIs Across Various Solid Tumors

**Figure 1:**
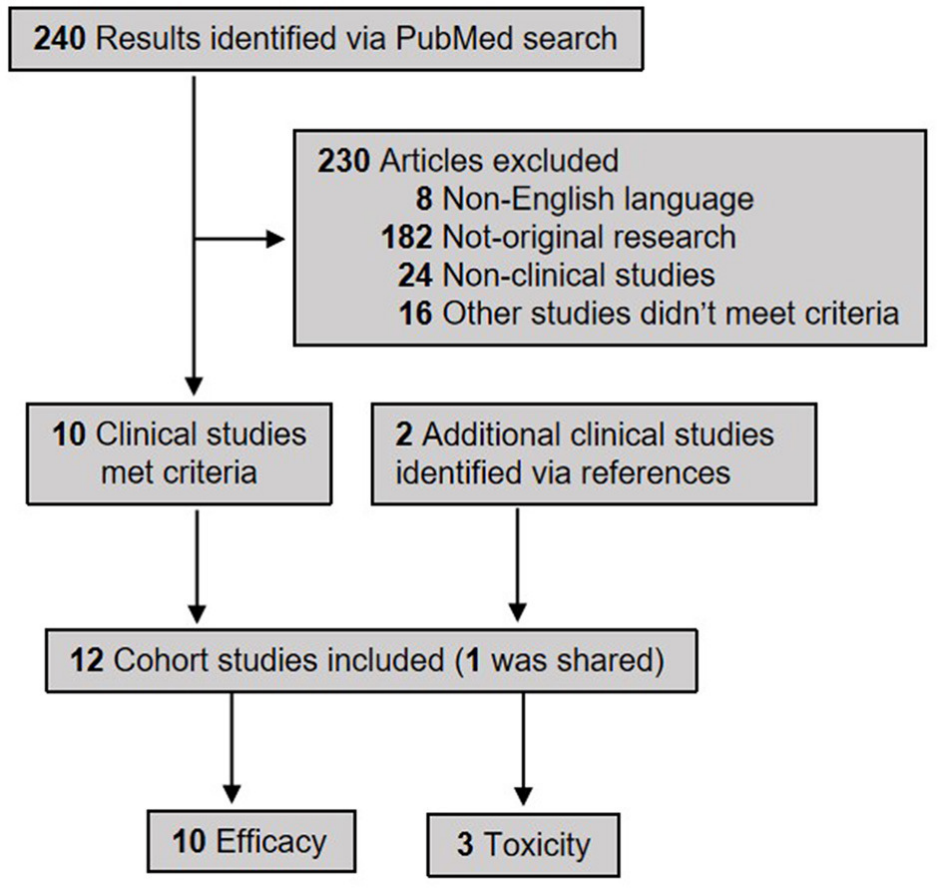
The PRISMA flow diagram of selecting publications to study the correlation of gut microbiome with the efficacy and adverse effects of ICIs across various solid tumors. In total 10 studies were included for the analysis of gut microbiome in correlating with the therapeutic efficacy of immunotherapy, and three studies for toxicity (adverse effects).

Using search keywords “gut microbiome” AND “cancer” AND “immunotherapy,” a total of 240 articles were retrieved from PubMed. With stringent manual screening and inclusion of two additional studies from the references, a total of 12 clinical studies were identified that meet our criteria to study the role of gut microbiome in cancer immunotherapy (Figure 1). The vast majority are prospective studies. Among them, 10 studies (6, 9, 12–19) had response/efficacy data and three studies (17, 20, 21) had AEs data using ICI therapy, and one study had both (17). Of note, the documented AEs in that three studies (17, 20, 21) were virtually all irAEs. The types of solid tumor involved include melanoma, non-small cell lung cancer (NSCLC), small cell lung cancer (SCLC), renal cell carcinoma (RCC) and hepatocellular carcinoma (HCC). There were 433 cancer patients (age range, 21-92 years-old) from four countries: USA (four studies), France (three studies), China (two studies), and Japan (one study) included in studies relevant to therapeutic response/efficacy; and 86 subjects (age range, 28-85 years-old) from the countries of USA, France and China included in studies relevant to the AEs of ICI treatment.

**Figure 2:**
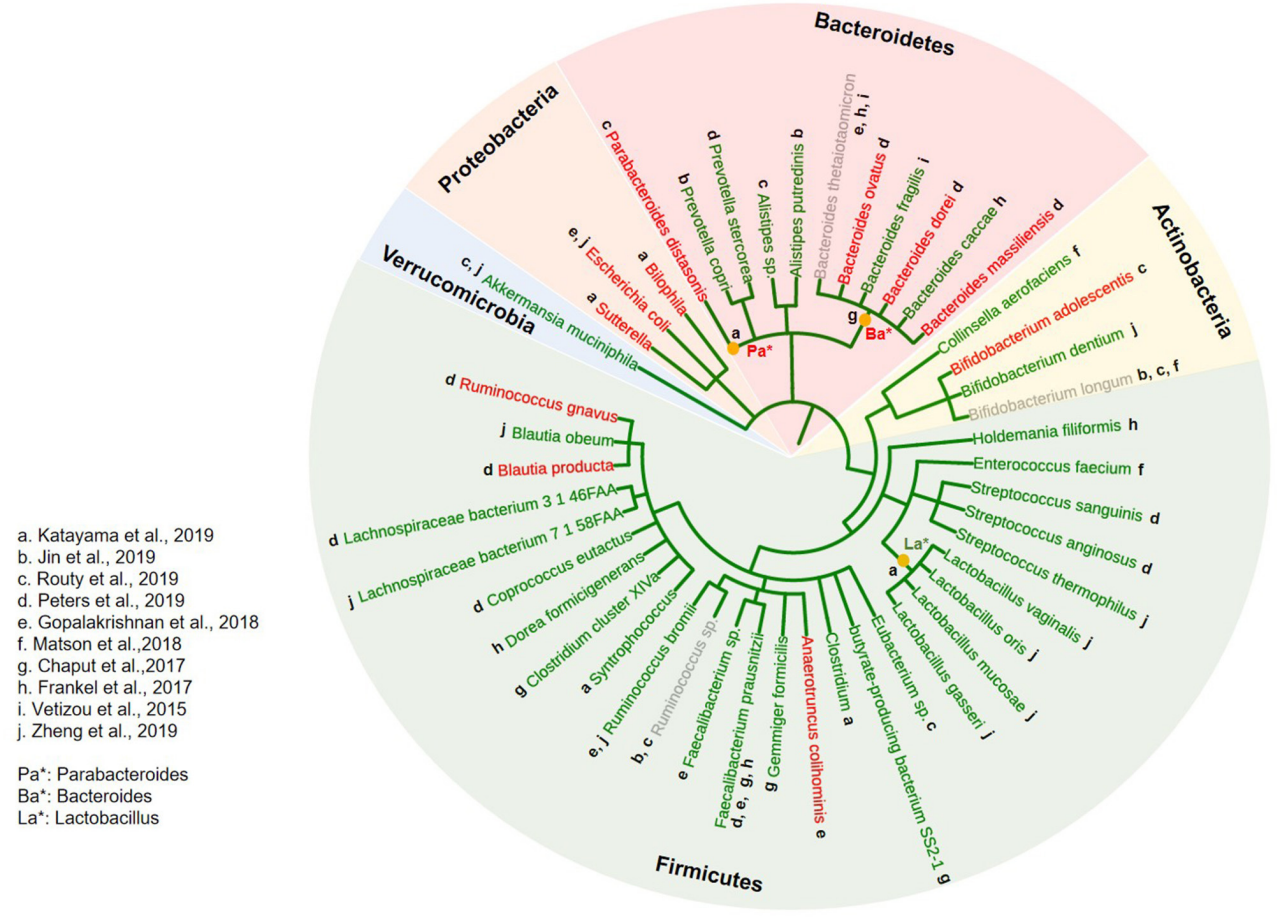
Correlation of gut microbiome to the treatment response of ICIs across various solid tumors. A phylogenetic tree was constructed using the phyloT software (https://phylot.biobyte.de) to capture and categorize all bacterial taxa reported to be associated with the treatment response from ICI in clinical studies across various solid tumors, ranking from phylum to species inside-out. Bacteria correlated to better response were labeled in green, and poor response in red. Those with mixed reports were labeled in gray. The lowercase alphabetical letters next to each bacterium indicate the individual studies from which bacterial taxa information was derived. The asterisks (*) indicate identified bacteria taxa at the genus level.

We extracted the taxa data of gut microbiome and plotted on phyloT. As shown in Figure 2, at the phylum level, it is clear that the enrichment of *Firmicutes* and *Verrucomicrobia* are correlated with better clinical outcome (labeled in green; related to better treatment response; and/or longer survival), whereas increased abundance in *Proteobacteria* was clearly associated with poor response (labeled in red). Although enrichment of *Bacteroidetes* correlated to poor response in some studies, opposite association and contradictory findings (labeled in gray) were also noticed in some other studies. Similarly, a mixed association of *Actinobacteria* to ICI treatment response was noticed.

**Figure 3:**
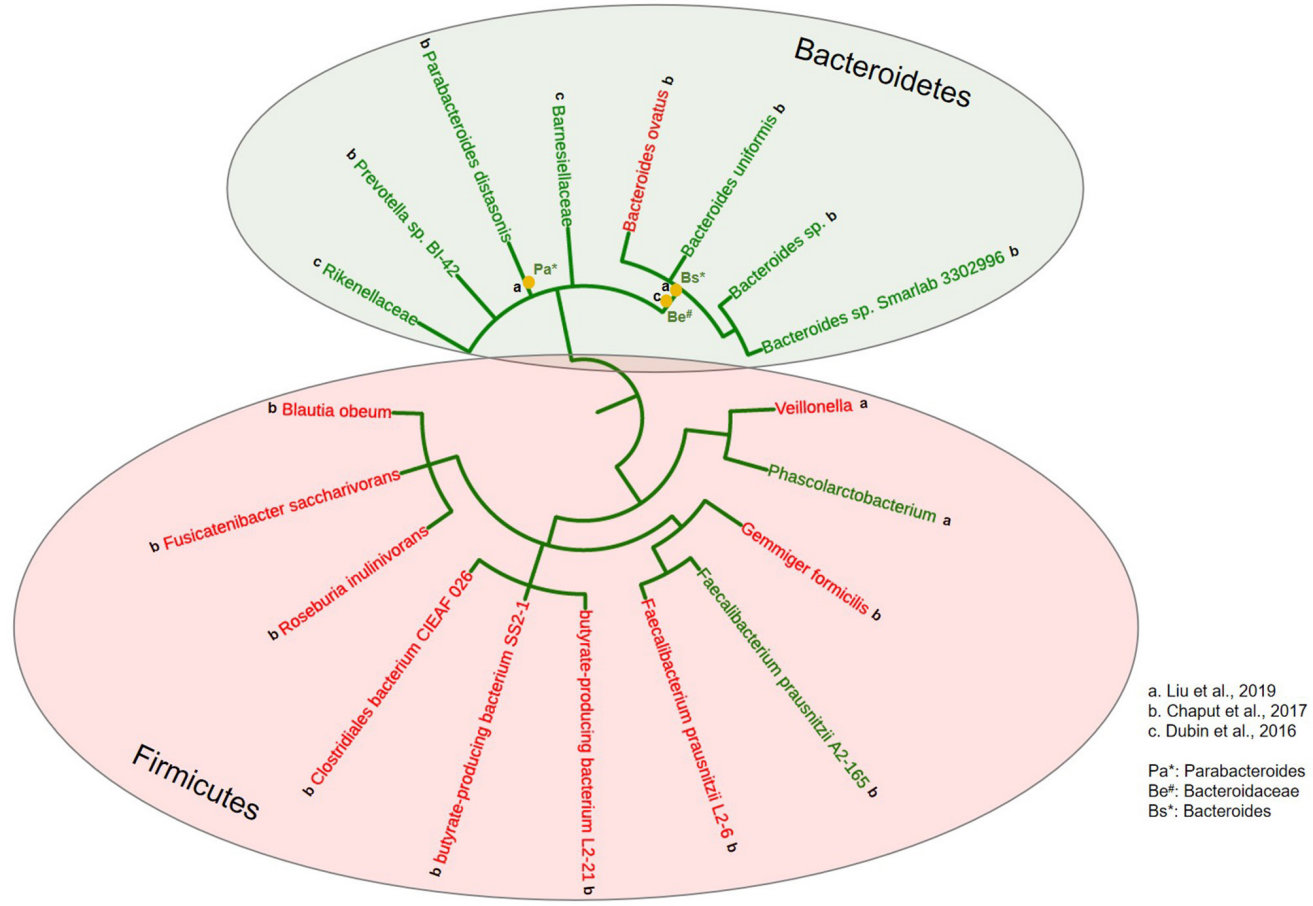
Correlation of gut microbiome to the toxicity of ICIs across various solid tumors. A phylogenetic tree was constructed using the phyloT software (https://phylot.biobyte.de) to capture and categorize all bacterial taxa reported to be associated with the adverse effects from ICI treatment in clinical studies across various solid tumors, ranking from phylum to species inside-out. Bacteria correlated to less toxicity were labeled in green, and more toxicity in red. The lowercase alphabetical letters next to each bacterium indicate the individual studies from which bacterial taxa information was derived. The asterisks (*) indicate identified bacteria taxa at the genus level.

However, regarding the potential link of gut microbiome to the AEs from ICIs, we noticed that the enrichment of *Firmicutes* interestingly correlated to higher incidence of AEs (essentially all irAEs, colored in red). This is reminiscent of clinical observations that patients who develop ICI AEs seem to have better treatment response (22). In contrast, *Bacteroidetes*, which is believed to be associated with less response, also correlated to less AEs (labeled in green, Figure 3).

### The Potential Impact of Antibiotics on the Therapeutic Effect of ICIs

Noticing the association of gut microbiome with ICI treatment response, we questioned if antibiotics, as potent modifiers of gut microbiota, could potentially affect the treatment response from ICIs. Using search keywords “antibiotics” AND “immunotherapy” AND “microbiome” AND “cancer,” we identified 17 eligible studies (Supplementary Figure 1) including two prospective (23, 24) and 15 retrospective studies (9–11, 25–36). There were in total 2,593 participants with various solid tumors including lung cancer, melanoma, RCC, HCC, colorectal cancer, head and neck cancer, bladder cancer, gastric cancer, esophageal cancer, cervical cancer, and others. Among them, 29.9% (775) of them received antibiotics treatment, 15 out of 17 received broad-spectrum antibiotics while two did not report the types of antibiotics.

**Figure 4:**
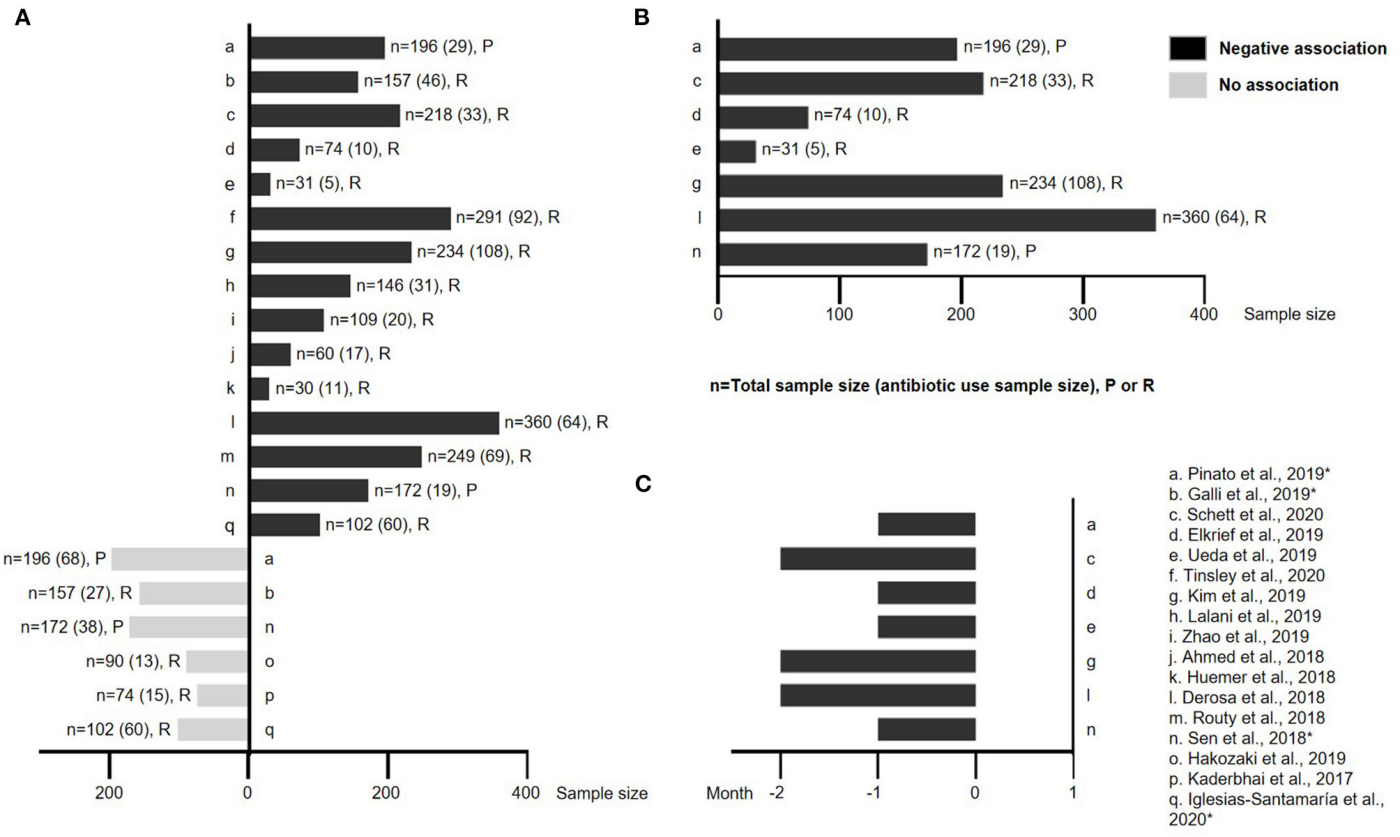
The impact of antibiotic exposure on ICI treatment across various solid tumors. **(A)** A schematic illustration showing studies with either negative or no association between antibiotic use and clinical outcome from ICI treatment. The study name, sample size and retrospective vs. prospective nature are all labeled. **(B)** Studies (including both retrospective and prospective) that have antibiotic use within 2 months prior to the initiation of ICI treatment were universally associated with poor clinical outcome. **(C)** Detail timing and duration of antibiotic use for studies shown in **(B)**. **n**: sample size; **P**: prospective study; **R**: retrospective study; *: mixed results based on the timing of antibiotic use.

As shown in Figure 4A, majority of these studies supported the hypothesis that the use of antibiotics has negative impact on the clinical outcome in patients receiving ICI treatment. However, there were also a few studies that suggested no obvious association or impact. Interestingly, two prospective studies (23, 24) and one retrospective study (25) provided seemingly different results (negative vs. no impact) when different timing of antibiotic exposure was put into consideration, suggesting that the timing and possibly the duration of antibiotics during ICI treatment are potentially important and will need further studies to clarify its impact.

In order to validate this hypothesis, we isolated the effect of the timing and duration of antibiotic exposure from all studies. Supplementary Figure 3 showed individual studies that exhibited either negative (labeled with black bars) or no association (labeled in gray bars) with ICI treatment. Among them, two studies (23, 24) were prospective (labeled with ^*^). Across all studies, it clearly demonstrated that only antibiotic exposure within 2 months prior to the initiation of ICIs universally exhibited negative impact on treatment response of ICIs (Figures 4B,C), except one study (10) (Supplementary Figure 3).

### Diet Could Potentially Affect the Efficacy of Cancer Immunotherapy

Using search keywords “diet” or “nutrition,” “microbiome,” “cancer” and “immunotherapy,” and their combinations, we were not able to extract sufficient number of clinical studies that directly link diet to cancer immunotherapy, including those published in abstract format (37), which is suggestive of an unmet need in this area. Since gut microbiome impacts cancer immunotherapy, we then investigated whether diet will have effect on gut microbiome that could potentially affect cancer immunotherapy. Based on Figure 2 and published data, here we define “ICI-favoring” diet as those that enrich *Firmicutes* or *Verrucomicrobia*, or reduce the abundance of *Proteobacteria*, or increase α diversity in gut microbiota, and the “ICI-unfavoring” diet as those that have the opposite effects.

To minimize confounding factors (especially various disease status), we used search terms “diet” AND “gut microbiome” AND “healthy adult” and included only clinical studies in healthy participants that have detailed diet and gut microbiome information (Supplementary Figure 2). We identified 16 eligible clinical studies (38–53) that included in total 771 subjects. Among them, 428 were females and 343 were males. Their age ranged 18–72.4 years and BMI ranged 19–36.6 kg/m^2^. These clinical studies were conducted in five countries including USA, China, Germany, UK and Belgium. We broadly categorized diet into plant-based diet which mainly contained whole grain, brassica vegetables, walnut and almond, etc; and animal-based diet which used red meat, animal fat and cheese, etc. There are only four studies using animal-based diet (40, 41, 47, 53). Although they also contained non-animal-based diet component, we were able to precisely derive data that are only relevant to animal-based diet.

**Figure 5:**
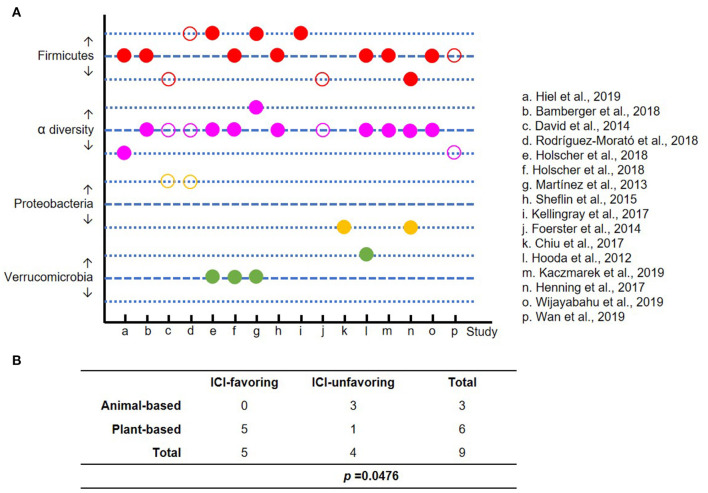
The impact of dietary intervention on gut microbiome. To minimize the confounding factors, only studies on healthy adults were included. **(A)** The alterations of gut microbiome after dietary intervention are displayed in 3 lines, which represent increase, no change and decrease in each category (red: *Firmicutes*; purple: α diversity; orange: *Proteobacteria*; and green: *Verrucomicrobia*). Solid and hollow circles represent plant- and animal-based diet, respectively. **(B)** A Fisher's exact test to compare the effect of plant- vs. animal-based diet on the enrichment of “ICI-favoring” vs. “ICI-unfavoring” gut microbiota (*P* = 0.0476).

Figure 5A in each category, depicts increase or decrease in relative abundance of *Firmicutes* or *Verrucomicrobia* or *Proteobacteria* or α diversity with demonstration of corresponding plant-based diet (labeled as solid dot) and animal-based diet (labeled as hollow circle), respectively. Using above defined “ICI-favoring” and “ICI-unfavoring” criteria, we found that three animal-based diet studies were “ICI-unfavoring” and none were “ICI-favoring.” Among the 12 plant-based diet studies, we found five were “ICI-favoring” and 1 was “ICI-unfavoring.” In summary, plant-based diet is found to be significantly associated with “ICI-favoring” gut microbiome, whereas animal-based diet is the opposite (Figure 5B, *p* = 0.0476). Diet studies that have mixed association, for example a reduced abundance in both the *Firmicutes* and *Proteobacteria* as shown in study n (51) in Figure 5A, were not included in the statistical analysis. We have also looked into various dietary patterns such as Mediterranean diet, Western diet, high-fiber diet, etc., however we were able to identify only very few relevant studies for us to draw meaningful conclusions.

## Discussion

Despite the great success of cancer immunotherapy using ICIs, their therapeutic benefits are limited by either various resistance mechanisms (54) or irAEs (3). Gut microbiome, due to its proven role in cancer development and immune regulation, has gained increasing expectation as a potential armamentarium to further improve cancer immunotherapy. It is speculated that gut microbiota could potentially affect the efficacy of ICIs through the modulation of immune checkpoints expression; dendritic cell function; lymphocyte homing, circulation and recruitment; as well as the production of critical metabolites such as short chain fatty acids (SCFA), etc. (55, 56). Furthermore, gut microbiota could influence host systemic immunity via cytokine secretion, primed lymphocyte circulation and antigen cross-reactivation induced tissue targeting (56). In consistent with this, we have recently shown that certain gut microbiota correlates significantly to ICI response in NSCLC patients (8), which echoes the findings from other groups (9, 15, 16), as well as preclinical mouse models (7). More importantly, a very recent phase 1 trial has demonstrated fecal microbiota transplantation (FMT) promoted response in ICI-refractory melanoma patients, which was associated with favorable changes in both the gut and tumor microenvironment (57). However, to better harness gut microbiome for clinical applicability, we need to understand whether there is shared gut microbiome feature across various solid tumors treated with ICIs, and whether common gut microbiome modifiers could have impact on ICI therapy.

Using series of systematic review, we first noticed that the enrichment of *Firmicutes* clearly correlated with better ICI response across various solid tumors. This is consistent with a previous report from Gopalakrishnan et al. whose work covered both solid and hematologic tumors (58). In addition, the reciprocal changes in abundance of *Verrucomicrobia* and *Proteobacteria* respectively, was found associated with better ICI response. Although further mechanistic studies are warranted to explain such observations, some speculated that the positive association of *Firmicutes* could in part due to their critical role in producing SCFA, a metabolite that has regulatory effect on inflammation and T cell differentiation (59–61). This is especially true for the fermentation of fiber to SCFA as the necessary enzymatic processes involved, which are largely dependent upon bacteria within the *Clostridia* class in the *Firmicutes* phylum (62). In agreement with this, a recent clinical study demonstrated that elevated fecal SCFA concentration significantly correlates with better clinical outcome from anti-PD-1 treatment across various solid tumors (63). This may also explain the positive correlation of mucin-degrading bacteria *Akkermansia muciniphila* (phylum *Verrucomicrobia*) to ICI response since it produces SCFA (both propionate and acetate) (64, 65). The negative association of *Proteobacteria* with ICI response is likely due to its close link to dysbiosis (66), which could account for the immune dysfunction in some non-responders to ICI therapy (5, 9, 15).

Although there are studies correlating phylum *Bacteroidetes* with poor response from ICIs (12, 17), we also observed several other studies were suggestive of a positive impact (6, 13, 18). In fact, an earlier preclinical study demonstrated a cause and effect role of certain *Bacteroidetes* (e.g., *B. thetaiotaomicron* or *B. fragilis*) in enhancing the therapeutic effect of anti-CTLA-4 agent (6). In addition, *Bacteroidetes* was found to digest insoluble fibers and mucins and provide SCFA and other metabolites to *Firmicutes*, suggesting its supporting role (67). This is consistent with a recent study using 11 bacteria strain mixture (11-mix: 7 *Bacteroidetes*, 3 *Firmicutes* and 1 *Fusobacteria*): when inoculated into germ-free mice, the 7 *Bacteroidetes*-mix failed to induce IFNγ^+^ CD8 T cells, whereas the 4-mix (3 *Firmicutes* and 1 *Fusobacteria*) displayed a significantly better induction capacity. However, the 4-mix alone was not sufficient to achieve the full inductive effect of the 11-mix for a maximal anti-cancer immunity (68). Interestingly, in our study, *Bacteroidetes* enrichment clearly correlated with less ICI-induced toxicity whereas *Firmicutes* abundance was obviously linked to increased incidence of irAEs. While such finding is reminiscent of our clinical observation that patients who experience greater irAEs tend to have better response from ICIs (22), it also supports the concept of using appropriate mix of *Firmicutes* and *Bacteroidetes* to enhance immunotherapy response yet mitigate irAEs (68).

As the ICI-favoring *Firmicutes* are the dominant gut microbial phyla, it is not surprising to see a negative impact on ICIs with the use of broad-spectrum antibiotics as *Firmicutes* will likely be affected most. In addition, antibiotics can induce dysbiosis (69). Our study has demonstrated that the timing and/or duration of antibiotics are critical, which probably explains the discrepancy observed in different studies, as certain period of time is required for gut microbiome to recover after antibiotics exposure. Interestingly, a recent study in healthy adults found that it took about 1.5 months for the gut microbiota of the subjects to recover to near-baseline composition after 4-day intervention with a cocktail of three antibiotics: meropenem, gentamicin and vancomycin (70). This finding is a perfect match to what we have observed in this study that antibiotics exposures within 2 months prior to the initiation of ICIs were universally associated with poor clinical outcome (23, 24, 26–28, 30, 35). In consistent with this, using a more quantitative analysis, Wilson B et al. found through their meta-analysis that the negative impact of antibiotics on the overall survival of patients with solid malignancies treated with ICI was greatest when antibiotic exposures was within 42 days prior to the initiation of ICI (71). Since antibiotic use is quite common among cancer patients, it will be interesting to see whether narrow-spectrum antibiotics could have selective effect on ICI response, especially considering the vast majority of *Firmicutes* bacteria are Gram-positive. In addition, if the use of antibiotics is inevitable, it will be important to understand whether the use of pre- and probiotics will have protective value under this situation.

Since diet is considered as a pivotal determinant of gut microbiota community among various host-endogenous and host-exogenous factors, we sought to determine its impact on ICIs. Despite the lack of direct evidence, we did observe that plant-based diet enriched “ICI-favoring” gut microbiome, represented as increased *Firmicutes* or *Verrucomicrobia* or α diversity, or reduced abundance of *Proteobacteria*. Such finding is consistent with a recent study on melanoma patients demonstrating that the response to immunotherapy can be influenced by dietary manipulation (37)—patients who consumed a high-fiber diet (plant-based) were about five times as likely to respond to anti–PD-1 treatment compared to patients who consumed a low-fiber diet (37). Further studies are warranted to clarify the potential value of diet/nutrition in both the treatment response and irAEs of cancer immunotherapy. In addition, it will be critically important to understand how particular diet affects gut microbiota and its metabolites before it can be better harnessed to modulate gut microbiome and its impact on cancer immunotherapy.

This study is based on systematic literature review, and therefore it is retrospective in nature. In addition, it is subject to selection bias, for example only original articles in English that are published on PubMed were used.

## Conclusions

There is shared feature of gut microbiome that correlates with the outcome of immunotherapy across various solid tumors, which can be potentially affected by antibiotics and diet. Further mechanistic studies are warranted to clarify their role to better harness gut microbiome for the improvement of cancer immunotherapy.

## Data Availability Statement

The raw data supporting the conclusions of this article will be made available by the authors, without undue reservation.

## Author Contributions

JZ conceived and designed the study, analyzed the data, guided essay writing, and provided critical revisions for this manuscript. CheH conducted database searches, extracted and analyzed the data, drew the figures, and provided the initial draft and prepared the manuscript. ML, BL, and HZ contributed to vital assistance in analyzing data and generating the figures. QD, XF, KM, ChaH, PN, FW, WS, SU, and CZ provided the consultation and critical revision of the manuscript for important intellectual content. All authors read and approved the final manuscript.

## Conflict of Interest

The authors declare that the research was conducted in the absence of any commercial or financial relationships that could be construed as a potential conflict of interest. The handling editor declared a past co-authorship with one of the authors JZ.

## Abbreviations

AEs: adverse effects
ICIs: immune checkpoint inhibitors
irAEs: immune-related AEs
NSCLC: non-small cell lung cancer
SCLC: small cell lung cancer
RCC: renal cell carcinoma
HCC: hepatocellular carcinoma
FMT: fecal microbiota transplantation
SCFA: short chain fatty acids.
