# Supplementary material for: Relating Gut Microbiome and Its Modulating Factors to Immunotherapy in Solid Tumors: A Systematic Review
Source: Front Oncol. 2021 Mar 18;11:642110. doi: 10.3389/fonc.2021.642110 (PMC8012896; doi:10.3389/fonc.2021.642110)

Supplemental Figure 1

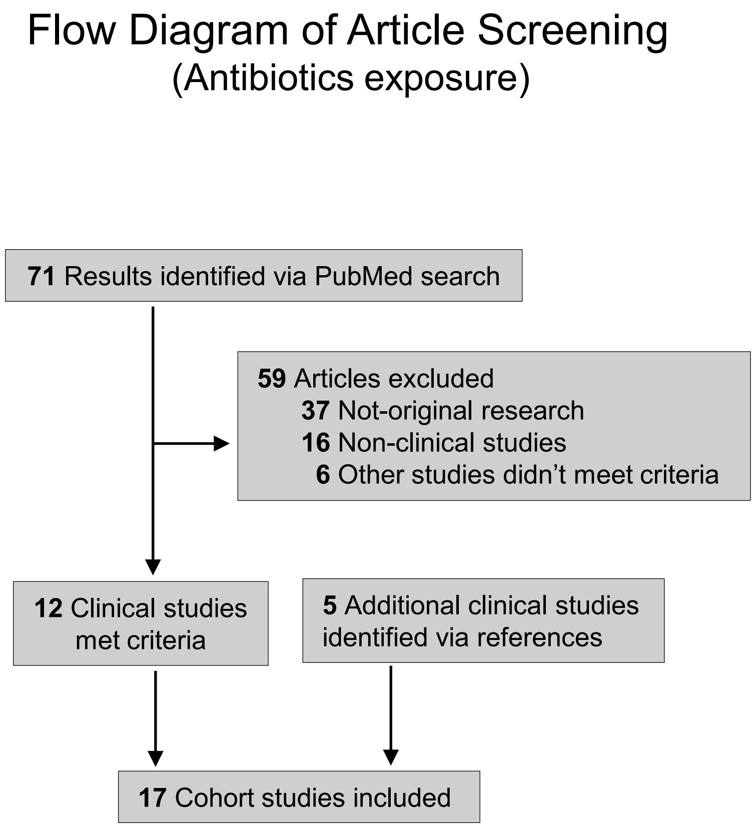

Supplemental Figure 2

Flow Diagram of Article Screening
(Dietary intervention)

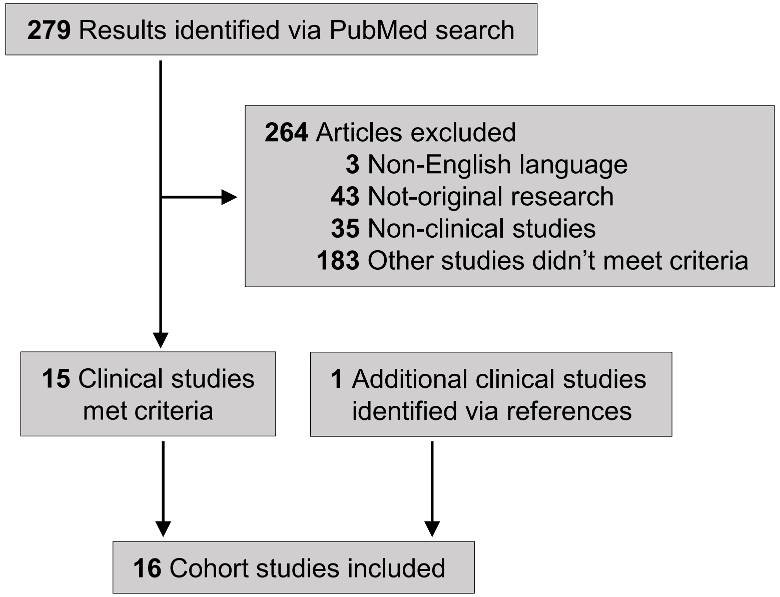

Supplemental Figure 3

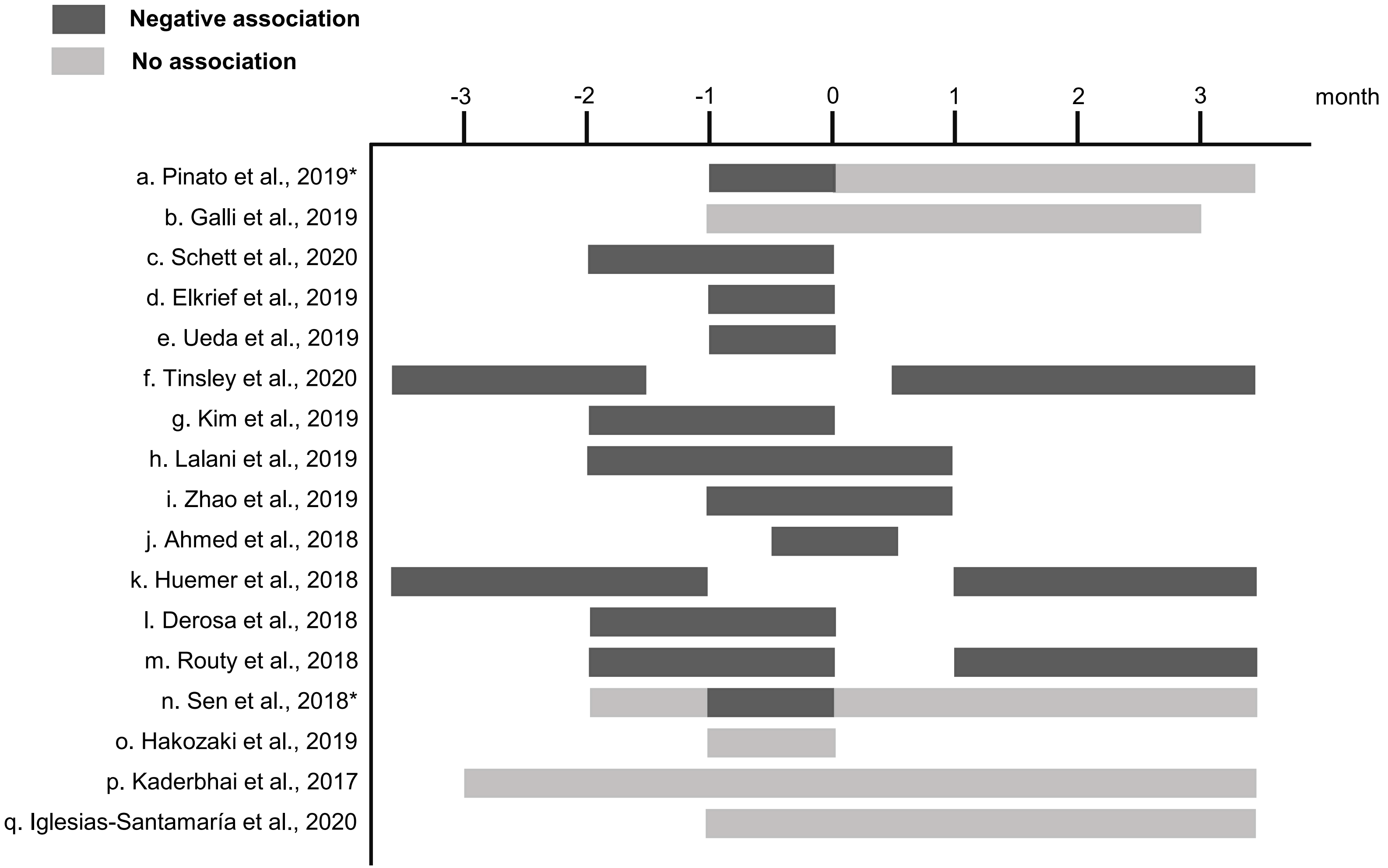

Supplement: Supplementary file 1 [file Data_Sheet_1.PDF]
