# Supplementary material for: Relating Gut Microbiome and Its Modulating Factors to Immunotherapy in Solid Tumors: A Systematic Review
Source: Front Oncol. 2021 Mar 18;11:642110. doi: 10.3389/fonc.2021.642110 (PMC8012896; doi:10.3389/fonc.2021.642110)
Supplement: Supplementary file 2 [file Data_Sheet_2.PDF]

Literature for “Response and irAEs”

Serial number	PMID	Year	Property	Classification	Note 1	Note 2
1	29634945	2018	Review 1	Not-original research		
2	29097494	2018	Selected article	Cohort studies included	Figure 2: Study c	
3	30684593	2019	Review 2	Not-original research		
4	29097493	2018	Selected article	Cohort studies included	Figure 2: Study e	
5	29798856	2018	Fundamental research	Non-clinical studies		
6	30842679	2019	Review 3	Not-original research		
7	29567708	2018	Review 4	Not-original research		
8	28270698	2017	Review 5	Not-original research		
9	29302014	2018	Selected article	Cohort studies included	Figure 2: Study f	
10	28303904	2017	Review 6	Not-original research		
11	31202124	2019	Review 7	Not-original research		
12	30755690	2019	Review 8	Not-original research		
13	27383982	2016	Review 9	Not-original research		
14	29567829	2018	Clinical study	Other studies didn't meet criteria		
15	29558948	2018	Review 10	Not-original research		
16	26541610	2015	Selected article	Cohort studies included	Figure 2: Study i	
17	26541606	2015	Fundamental research	Non-clinical studies		
18	27332730	2016	Review 11	Not-original research		
19	27607555	2016	Review 12	Not-original research		
20	30712808	2019	Review 13	Not-original research		
21	30995949	2019	Review 14	Not-original research		
22	30609850	2019	Review 15	Not-original research		
23	28722709	2017	Review 16	Not-original research		
24	31533218	2019	Review 17	Not-original research		
25	26970951	2016	Review 18	Not-original research		
26	30339501	2019	Review 19	Not-original research		
27	30458058	2019	Review 20	Not-original research		
28	31159348	2019	Review 21	Not-original research		
29	31087391	2020	Review 22	Not-original research		
30	31003463	2019	Review 23	Not-original research		
31	31450712	2019	Review 24	Not-original research		
32	28527946	2017	Review 25	Not-original research		
33	30803449	2019	Review 26	Not-original research		
34	29789015	2018	Review 27	Not-original research		
35	29217301	2018	Review 28	Not-original research		
36	30131322	2018	Review 29	Not-original research		
37	31099674	2019	Review 30	Not-original research		
38	30392721	2018	Review 31	Not-original research		
39	30280940	2018	Review 32	Not-original research		
40	30001289	2018	Review 33	Not-original research		
41	31634731	2019	Review 34	Not-original research		
42	31277279	2019	Review 35	Not-original research		
43	31337439	2019	Selected article	Cohort studies included	Figure 2: Study j	

<table>
<tr><td>44</td><td>31216061</td><td>2019</td><td>Review 36</td><td>Not-original research</td><td></td><td></td></tr>
<tr><td>45</td><td>31293523</td><td>2019</td><td>Review 37</td><td>Not-original research</td><td></td><td></td></tr>
<tr><td>46</td><td>31597596</td><td>2019</td><td>Review 38</td><td>Not-original research</td><td></td><td></td></tr>
<tr><td>47</td><td>30420754</td><td>2018</td><td>Clinical study</td><td>Other studies didn't meet criteria</td><td></td><td></td></tr>
<tr><td>48</td><td>30655087</td><td>2019</td><td>Review 39</td><td>Not-original research</td><td></td><td></td></tr>
<tr><td>49</td><td>29449660</td><td>2018</td><td>Review 40</td><td>Not-original research</td><td></td><td></td></tr>
<tr><td>50</td><td>29580257</td><td>2018</td><td>Review 41</td><td>Not-original research</td><td></td><td></td></tr>
<tr><td>51</td><td>30387230</td><td>2018</td><td>Fundamental research</td><td>Non-clinical studies</td><td></td><td></td></tr>
<tr><td>52</td><td>28124291</td><td>2017</td><td>Review 42</td><td>Not-original research</td><td></td><td></td></tr>
<tr><td>53</td><td>29893261</td><td>2018</td><td>Review 43</td><td>Not-original research</td><td></td><td></td></tr>
<tr><td>54</td><td>29610286</td><td>2018</td><td>Commentary</td><td>Not-original research</td><td></td><td></td></tr>
<tr><td>55</td><td>29379189</td><td>2018</td><td>Review 44</td><td>Not-original research</td><td></td><td></td></tr>
<tr><td>56</td><td>31109338</td><td>2019</td><td>Review 45</td><td>Not-original research</td><td></td><td></td></tr>
<tr><td>57</td><td>31014119</td><td>2019</td><td>Review 46</td><td>Not-original research</td><td></td><td></td></tr>
<tr><td>58</td><td>32309602</td><td>2018</td><td>Review 47</td><td>Not-original research</td><td></td><td></td></tr>
<tr><td>59</td><td>31308831</td><td>2019</td><td>Review 48</td><td>Not-original research</td><td></td><td></td></tr>
<tr><td>60</td><td>31344431</td><td>2019</td><td>Review 49</td><td>Not-original research</td><td></td><td></td></tr>
<tr><td>61</td><td>31026576</td><td>2019</td><td>Selected article</td><td>Cohort studies included</td><td>Figure 2: Study b</td><td></td></tr>
<tr><td>62</td><td>30510057</td><td>2018</td><td>Review 50</td><td>Not-original research</td><td></td><td></td></tr>
<tr><td>63</td><td>31340438</td><td>2019</td><td>Review 51</td><td>Not-original research</td><td></td><td></td></tr>
<tr><td>64</td><td>30887236</td><td>2019</td><td>Review 52</td><td>Not-original research</td><td></td><td></td></tr>
<tr><td>65</td><td>31517538</td><td>2019</td><td>Review 53</td><td>Not-original research</td><td></td><td></td></tr>
<tr><td>66</td><td>29631891</td><td>2018</td><td>Review 54</td><td>Not-original research</td><td></td><td></td></tr>
<tr><td>67</td><td>31046972</td><td>2019</td><td>Review 55</td><td>Not-original research</td><td></td><td></td></tr>
<tr><td>68</td><td>29437871</td><td>2018</td><td>Translational research</td><td>Non-clinical studies</td><td></td><td></td></tr>
<tr><td>69</td><td>31663125</td><td>2020</td><td>Review 56</td><td>Not-original research</td><td></td><td></td></tr>
<tr><td>70</td><td>30615894</td><td>2019</td><td>Review 57</td><td>Not-original research</td><td></td><td></td></tr>
<tr><td>71</td><td>29113654</td><td>2017</td><td>Review 58</td><td>Not-original research</td><td></td><td></td></tr>
<tr><td>72</td><td>30939293</td><td>2019</td><td>Review 59</td><td>Not-original research</td><td></td><td></td></tr>
<tr><td>73</td><td>31597568</td><td>2019</td><td>Selected article</td><td>Cohort studies included</td><td>Figure 2: Study d</td><td rowspan="3">2 additional clinical studies from the Reference 11, 12
Reference 11: (Figure 2: Study h)
Reference 12: (Figure 2: Study g and Figure 3: b)</td></tr>
<tr><td>74</td><td>29420293</td><td>2018</td><td>Translational research</td><td>Non-clinical studies</td><td></td></tr>
<tr><td>75</td><td>31175337</td><td>2019</td><td>Review 60</td><td>Not-original research</td><td></td></tr>
<tr><td>76</td><td>31142858</td><td>2019</td><td>Translational research</td><td>Non-clinical studies</td><td></td><td></td></tr>
<tr><td>77</td><td>30914517</td><td>2019</td><td>Japanese</td><td>Non-English language</td><td></td><td></td></tr>
<tr><td>78</td><td>30190370</td><td>2018</td><td>Fundamental research</td><td>Non-clinical studies</td><td></td><td></td></tr>
<tr><td>79</td><td>31637019</td><td>2019</td><td>Review 61</td><td>Not-original research</td><td></td><td></td></tr>
<tr><td>80</td><td>31532533</td><td>2019</td><td>Review 62</td><td>Not-original research</td><td></td><td></td></tr>
<tr><td>81</td><td>31205532</td><td>2019</td><td>Review 63</td><td>Not-original research</td><td></td><td></td></tr>
<tr><td>82</td><td>32016028</td><td>2019</td><td>Commentary</td><td>Not-original research</td><td></td><td></td></tr>
<tr><td>83</td><td>32192986</td><td>2020</td><td>Review 64</td><td>Not-original research</td><td></td><td></td></tr>
<tr><td>84</td><td>30332668</td><td>2019</td><td>Review 65</td><td>Not-original research</td><td></td><td></td></tr>
<tr><td>85</td><td>30218667</td><td>2018</td><td>Review 66</td><td>Not-original research</td><td></td><td></td></tr>
<tr><td>86</td><td>27940475</td><td>2017</td><td>Review 67</td><td>Not-original research</td><td></td><td></td></tr>
<tr><td>87</td><td>29665135</td><td>2019</td><td>Clinical study</td><td>Other studies didn't meet criteria</td><td></td><td></td></tr>
<tr><td>88</td><td>29494275</td><td>2018</td><td>Commentary</td><td>Not-original research</td><td></td><td></td></tr>
<tr><td>89</td><td>28682227</td><td>2017</td><td>Review 68</td><td>Not-original research</td><td></td><td></td></tr>
</table>

90	27028619	2016	Review 69	Not-original research		
91	29467322	2018	Translational research	Non-clinical studies		
92	31138779	2019	Fundamental research	Non-clinical studies		
93	29336431	2018	Commentary	Not-original research		
94	31427405	2019	Clinical study	Other studies didn't meet criteria		
95	30905818	2019	Review 70	Not-original research		
96	31209849	2019	A chapter of a book	Not-original research		
97	31680787	2019	Review 71	Not-original research		
98	32067144	2020	Review 72	Not-original research		
99	31831639	2019	Fundamental research	Non-clinical studies		
100	31827982	2019	Review 73	Not-original research		
101	29437869	2018	Review 74	Not-original research		
102	31268133	2019	Review 75	Not-original research		
103	31138229	2019	Introduction of study protocol	Other studies didn't meet criteria		
104	29735917	2018	Review 76	Not-original research		
105	29391185	2018	Review 77	Not-original research		
106	31616428	2019	Review 78	Not-original research		
107	31263961	2019	Review 79	Not-original research		
108	31519210	2019	Clinical study	Other studies didn't meet criteria		
109	26837003	2016	Selected article	Cohort studies included	Figure 3: Study c	
110	31530405	2019	Spanish	Non-English language		
111	31311009	2020	Review 80	Not-original research		
112	30935126	2019	Review 81	Not-original research		
113	32010123	2020	Review 82	Not-original research		
114	31107965	2019	Review 83	Not-original research		
115	31256356	2019	Clinical study	Other studies didn't meet criteria		
116	30652093	2018	Commentary	Not-original research		
117	31954488	2020	Review 84	Not-original research		
118	31073691	2019	Review 85	Not-original research		
119	31827379	2019	Review 86	Not-original research		
120	30848738	2019	Review 87	Not-original research		
121	30512251	2018	Review 88	Not-original research		
122	30173562	2018	Review 89	Not-original research		
123	30393044	2018	Highlights from annual meeting	Not-original research		
124	31701169	2019	Review 90	Not-original research		
125	27474734	2016	Review 91	Not-original research		
126	29746836	2018	Fundamental research	Non-clinical studies		
127	30845114	2019	Review 92	Not-original research		
128	32010563	2019	Selected article	Cohort studies included	Figure 2: Study a	
129	28844794	2017	Review 93	Not-original research		
130	31896938	2019	Review 94	Not-original research		
131	29129446	2017	News	Not-original research		
132	31450659	2019	Review 95	Not-original research		
133	30936499	2019	Review 96	Not-original research		
134	29891391	2018	Commentary	Not-original research		
135	30907164	2019	Review 97	Not-original research		

136	27806346	2017	Review 98	Not-original research		
137	30855275	2019	Fundamental research	Non-clinical studies		
138	30719076	2019	Review 99	Not-original research		
139	30365127	2019	Review 100	Not-original research		
140	30258117	2018	Fundamental research	Non-clinical studies		
141	30940601	2019	Review 101	Not-original research		
142	31214189	2019	Fundamental research	Non-clinical studies		
143	27210745	2016	Fundamental research	Non-clinical studies		
144	24778636	2014	Review 102	Not-original research		
145	29988102	2018	Clinical study	Other studies didn't meet criteria		
146	31281535	2019	Fundamental research	Non-clinical studies		
147	30258436	2018	Review 103	Not-original research		
148	30201858	2018	Communication	Not-original research		
149	29150490	2018	Meeting paper	Not-original research		
150	30282828	2018	Translational research	Non-clinical studies		
151	30183502	2019	Commentary	Not-original research		
152	28551360	2017	Review 104	Not-original research		
153	32301026	2020	A chapter of a book	Not-original research		
154	30305604	2018	Fundamental research	Non-clinical studies		
155	30892954	2019	Editorial	Not-original research		
156	29355853	2018	Commentary	Not-original research		
157	31345329	2019	Commentary	Not-original research		
158	30604388	2019	Review 105	Not-original research		
159	30940203	2019	Commentary	Not-original research		
160	31869338	2019	Clinical study	Other studies didn't meet criteria		
161	30786013	2019	Review 106	Not-original research		
162	32130695	2020	Review 107	Not-original research		
163	30324425	2019	Review 108	Not-original research		
164	30209679	2018	Review 109	Not-original research		
165	31884204	2020	Review 110	Not-original research		
166	30885328	2019	Clinical study	Other studies didn't meet criteria		
167	30906663	2019	Clinical study	Other studies didn't meet criteria		
168	32117767	2020	Review 111	Not-original research		
169	30489072	2019	Review 112	Not-original research		
170	31678050	2020	Review 113	Not-original research		
171	29691222	2018	Commentary	Not-original research		
172	31737072	2019	Review 114	Not-original research		
173	30583753	2018	Review 115	Not-original research		
174	29899754	2018	Editorial	Not-original research		
175	31292268	2020	Clinical study	Other studies didn't meet criteria		
176	29781826	2018	Review 116	Not-original research		
177	30642560	2019	French	Non-English language		
178	29406499	2018	News	Not-original research		
179	30818030	2019	Review 117	Not-original research		
180	29107046	2018	Review 118	Not-original research		
181	32112046	2020	Review 119	Not-original research		

182	31833336	2019	Editorial	Not-original research		
183	29780391	2018	Review 120	Not-original research		
184	26101781	2015	Review 121	Not-original research		
185	29537705	2018	Fundamental research	Non-clinical studies		
186	30523317	2018	Commentary	Not-original research		
187	32132244	2020	Review 122	Not-original research		
188	30003334	2018	Review 123	Not-original research		
189	30135516	2018	Review 124	Not-original research		
190	31904155	2020	Review 125	Not-original research		
191	32297948	2020	Clinical study	Other studies didn't meet criteria		
192	30632784	2020	Review 126	Not-original research		
193	31582175	2020	Review 127	Not-original research		
194	32475526	2020	A chapter of a book	Not-original research		
195	28008838	2016	Review 128	Not-original research		
196	29411013	2018	News & Analysis	Not-original research		
197	29556232	2018	Review 129	Not-original research		
198	32014010	2020	Clinical study	Other studies didn't meet criteria		
199	29176281	2017	Japanese	Non-English language		
200	31991820	2020	Fundamental research	Non-clinical studies		
201	29350031	2018	View	Not-original research		
202	29097525	2017	News	Not-original research		
203	30709565	2019	French	Non-English language		
204	29158588	2018	Commentary	Not-original research		
205	29795257	2018	News	Not-original research		
206	30980131	2019	Letter to the editor	Not-original research		
207	25812838	2015	Review 130	Not-original research		
208	31592683	2019	Editorial	Not-original research		
209	29702174	2018	Fundamental research	Non-clinical studies		
210	28100240	2017	Review 131	Not-original research		
211	31768581	2020	Translational research	Non-clinical studies		
212	25708215	2015	Review 132	Not-original research		
213	29251088	2017	Editorial	Not-original research		
214	31755554	2020	Fundamental research	Non-clinical studies		
215	30666802	2019	Clinical study	Other studies didn't meet criteria		
216	31914785	2020	Summary of annual meeting	Not-original research		
217	25170039	2014	Review 133	Not-original research		
218	26923000	2016	Review 134	Not-original research		
219	29509755	2018	News	Not-original research		
220	26612936	2015	Commentary	Not-original research		
221	32040356	2020	Review 135	Not-original research		
222	29614305	2018	Commentary	Not-original research		
223	26678336	2015	Commentary	Not-original research		
224	29217839	2018	Research Highlights	Not-original research		
225	28784244	2017	French	Non-English language		
226	31079502	2019	Review 136	Not-original research		
227	26687344	2015	View	Not-original research		

228	27379811	2016	Interview	Not-original research		
229	28336551	2017	Commentary	Not-original research		
230	32016358	2020	Clinical study	Other studies didn't meet criteria		
231	32116226	2020	Russian	Non-English language		
232	29982987	2018	Commentary	Not-original research		
233	30693820	2019	Selected article	Cohort studies included	Figure 3: Study a	
234	26875828	2016	Commentary	Not-original research		
235	27546223	2016	French	Non-English language		
236	26542545	2015	News	Not-original research		
237	28759258	2017	Review 137	Not-original research		
238	32460005	2020	Review	Not-original research		
239	28197360	2016	View	Not-original research		
240	32308598	2020	A case report	Non-clinical studies		

Literature for “Antibiotic exposure”

Serial number	PMID	Year	Property	Classification	Note 1	Note 2
1	29097494	2018	Selected article	Cohort studies included	Figure 4: Study m	
2	29567708	2018	Review 1	Not-original research		
3	24264989	2013	Fundamental research	Non-clinical studies		
4	26541610	2015	Clinical study	Other studies didn't meet criteria		
5	28270698	2017	Review 2	Not-original research		
6	31533218	2019	Review 3	Not-original research		
7	29789015	2018	Review 4	Not-original research		
8	28527946	2017	Review 5	Not-original research		
9	29449660	2018	Review 6	Not-original research		
10	29617710	2018	Selected article	Cohort studies included	Figure 4: Study l	
11	31427405	2019	Clinical study	Other studies didn't meet criteria		
12	31308831	2019	Review 7	Not-original research		
13	31142858	2019	Translational research	Non-clinical studies		
14	24264990	2013	Fundamental research	Non-clinical studies		
15	31634731	2019	Review 8	Not-original research		
16	29610286	2018	Commentary	Not-original research		
17	31046972	2019	Review 9	Not-original research		
18	31694714	2019	Commentary	Not-original research		
19	31268133	2019	Review 10	Not-original research		
20	31532533	2019	Review 11	Not-original research		
21	27940475	2017	Review 12	Not-original research		
22	31097097	2019	Selected article	Cohort studies included	Figure 4: Study b	
23	31236389	2019	Review 13	Not-original research		
24	29437871	2018	Translational research	Non-clinical studies		
25	31073691	2019	Review 14	Not-original research		
26	29467322	2018	Translational research	Non-clinical studies		
27	27210745	2016	Fundamental research	Non-clinical studies		
28	31352168	2019	Review 15	Not-original research		
29	32016028	2019	Commentary	Not-original research		Reference 11: (Figure 4: Study a)
30	31896938	2019	Review 16	Not-original research		
31	29746836	2018	Fundamental research	Non-clinical studies		
32	30305604	2018	Fundamental research	Non-clinical studies		
33	31741763	2019	Meta-analysis	Not-original research		
34	30257213	2018	Fundamental research	Non-clinical studies		
35	30885328	2019	Selected article	Cohort studies included	Figure 4: Study i	
36	31519210	2019	Clinical study	Other studies didn't meet criteria		
37	31954488	2020	Review 17	Not-original research		
38	30854072	2019	Selected article	Cohort studies included	Figure 4: Study o	
39	30183502	2019	Commentary	Not-original research		
40	31718585	2019	Selected article	Cohort studies included	Figure 4: Study g	
41	30906663	2019	Selected article	Cohort studies included	Figure 4: Study d	
42	30972292	2019	View	Not-original research		
43	31884204	2020	Review 18	Not-original research		

44	29858123	2018	Clinical study	Other studies didn't meet criteria		
45	29537705	2018	Fundamental research	Non-clinical studies		
46	31292268	2020	Selected article	Cohort studies included	Figure 4: Study f	3 additional clinical studies from the Reference 30, 31, 32<br>Reference 30: (Figure 4: Study k)<br>Reference 31: (Figure 4: Study j)<br>Reference 32: (Figure 4: Study n)
47	32173463	2020	A systematic review and meta-analysis 19	Not-original research		
48	24439901	2014	Fundamental research	Non-clinical studies		
49	30685590	2019	View	Not-original research		
50	30323090	2018	Clinical study	Other studies didn't meet criteria		
51	31865400	2020	Review 20	Not-original research		
52	32100550	2020	Review 21	Not-original research		
53	29777867	2018	Clinical study	Other studies didn't meet criteria		
54	28759258	2017	Review 22	Not-original research		
55	31745593	2020	Selected article	Cohort studies included	Figure 4: Study c	
56	26168477	2015	Review 23	Not-original research		
57	29340102	2017	Fundamental research	Non-clinical studies		
58	32008133	2020	Review 24	Not-original research		
59	30980131	2019	Letter to the editor	Not-original research		
60	31562048	2019	Selected article	Cohort studies included	Figure 4: Study h	
61	31919759	2020	Selected article	Cohort studies included	Figure 4: Study q	
62	28551664	2017	Selected article	Cohort studies included	Figure 4: Study p	
63	24504404	2014	Commentary	Not-original research		
64	23955389	2013	Fundamental research	Non-clinical studies		
65	29251088	2017	Editorial	Not-original research		
66	29449710	2018	News & Analysis	Not-original research		
67	32037950	2020	Review 25	Not-original research		
68	26678336	2015	Commentary	Not-original research		
69	24947042	2014	Fundamental research	Non-clinical studies		
70	28785009	2017	Fundamental research	Non-clinical studies		
71	21859957	2011	Fundamental research	Non-clinical studies		
	31704856	2019	Selected article	Cohort studies included	Figure 4: Study e	

Literature for “Dietary intervention”

Serial number	PMID	Year	Property	Classification	Note 1	Note 2
1	26269668	2015	Review 1	Not-original research		
2	22699609	2012	Clinical study	Other studies didn't meet criteria		
3	29489753	2018	Environmental research	Non-clinical studies		
4	26416813	2016	Clinical study	Other studies didn't meet criteria		
5	26100928	2016	Clinical study	Other studies didn't meet criteria		
6	31194939	2019	Clinical study	Other studies didn't meet criteria		
7	27272325	2016	Review 2	Not-original research		
8	26951067	2016	Clinical study	Other studies didn't meet criteria		
9	25758642	2015	Clinical study	Other studies didn't meet criteria		
10	31053143	2019	Clinical study	Other studies didn't meet criteria		
11	28936910	2017	Review 3	Not-original research		
12	31474424	2019	Review 4	Not-original research		
13	31108510	2019	Selected article	Cohort studies included	Figure 5: Study a	
14	30489399	2019	Review 5	Not-original research		
15	25584460	2015	Review 6	Not-original research		
16	28524627	2017	Review 7	Not-original research		
17	29470389	2018	Selected article	Cohort studies included	Figure 5: Study b	1 additional clinical study from the Reference 8<br>Reference 8: (Figure 5: Study c)
18	30479462	2018	Review 8	Not-original research		
19	30936547	2019	Meta-analysis	Not-original research		
20	27712080	2018	Review 9	Not-original research		
21	31291462	2019	Clinical study	Other studies didn't meet criteria		
22	26762459	2016	Fundamental research	Non-clinical studies		
23	25988339	2015	Clinical study	Other studies didn't meet criteria		
24	30612189	2019	Review 10	Not-original research		
25	30998992	2019	Fundamental research	Non-clinical studies		
26	27821203	2017	Review 11	Not-original research		
27	28614379	2017	Clinical study	Other studies didn't meet criteria		
28	30696735	2019	Clinical study	Other studies didn't meet criteria		
29	30425247	2018	Clinical study	Other studies didn't meet criteria		
30	30782617	2019	Clinical study	Other studies didn't meet criteria		
31	26514720	2015	Position and View	Not-original research		
32	27288567	2016	Clinical study	Other studies didn't meet criteria		
33	28988196	2018	Clinical study	Other studies didn't meet criteria		
34	30422704	2019	Translational research	Non-clinical studies		
35	20668239	2011	Clinical study	Other studies didn't meet criteria		
36	30991877	2019	Clinical study	Other studies didn't meet criteria		
37	28179226	2017	Clinical study	Other studies didn't meet criteria		
38	31043597	2019	Clinical study	Other studies didn't meet criteria		
39	29766369	2019	Review 12	Not-original research		
40	30836671	2019	Clinical study	Other studies didn't meet criteria		
41	29757343	2018	Review 13	Not-original research		
42	31051503	2019	Clinical study	Other studies didn't meet criteria		

43	29755475	2018	Clinical study	Other studies didn't meet criteria		
44	19043404	2009	Clinical study	Other studies didn't meet criteria		
45	29307330	2018	Clinical study	Other studies didn't meet criteria		
46	31258108	2019	Clinical study	Other studies didn't meet criteria		
47	27725146	2017	Clinical study	Other studies didn't meet criteria		
48	30888537	2019	Clinical study	Other studies didn't meet criteria		
49	29032502	2018	Review 14	Not-original research		
50	30550821	2019	Clinical study	Other studies didn't meet criteria		
51	28245817	2017	Fundamental research	Non-clinical studies		
52	29600282	2018	Clinical study	Other studies didn't meet criteria		
53	25851728	2015	Review 15	Not-original research		
54	30212253	2018	Clinical study	Other studies didn't meet criteria		
55	30231542	2018	Clinical study	Other studies didn't meet criteria		
56	31240835	2019	Clinical study	Other studies didn't meet criteria		
57	30636111	2019	Clinical study	Other studies didn't meet criteria		
58	28178201	2017	Clinical study	Other studies didn't meet criteria		
59	27719686	2016	Clinical study	Other studies didn't meet criteria		
60	30144429	2018	Clinical study	Other studies didn't meet criteria		
61	31317029	2019	Clinical study	Other studies didn't meet criteria		
62	29781841	2018	Review 16	Not-original research		
63	28986601	2018	Review 17	Not-original research		
64	31012086	2019	Clinical study	Other studies didn't meet criteria		
65	29867803	2018	Clinical study	Other studies didn't meet criteria		
66	30938383	2019	Clinical study	Other studies didn't meet criteria		
67	31136662	2019	Clinical study	Other studies didn't meet criteria		
68	30732197	2019	Clinical study	Other studies didn't meet criteria		
69	26562532	2015	Clinical study	Other studies didn't meet criteria		
70	30794085	2019	Clinical study	Other studies didn't meet criteria		
71	28213610	2017	Clinical study	Other studies didn't meet criteria		
72	26773784	2016	Clinical study	Other studies didn't meet criteria		
73	30661321	2019	Clinical study	Other studies didn't meet criteria		
74	30317146	2019	Selected article	Cohort studies included	Figure 5: Study m	
75	30698687	2019	Clinical study	Other studies didn't meet criteria		
76	31965839	2019	Fundamental research	Non-clinical studies		
77	31235863	2019	Clinical study	Other studies didn't meet criteria		
78	31582724	2019	Clinical study	Other studies didn't meet criteria		
79	28806487	2017	Clinical study	Other studies didn't meet criteria		
80	28526852	2017	Selected article	Cohort studies included	Figure 5: Study n	
81	31132957	2019	Clinical study	Other studies didn't meet criteria		
82	29267377	2017	Fundamental research	Non-clinical studies		
83	27381339	2016	Clinical study	Other studies didn't meet criteria		
84	29098426	2018	Clinical study	Other studies didn't meet criteria		
85	30867328	2019	Clinical study	Other studies didn't meet criteria		
86	29726951	2018	Selected article	Cohort studies included	Figure 5: Study e	
87	25453395	2015	Clinical study	Other studies didn't meet criteria		
88	22241860	2012	Clinical study	Other studies didn't meet criteria		

89	29925774	2018	Fundamental research	Non-clinical studies		
90	30827566	2019	Fundamental research	Non-clinical studies		
91	31722138	2019	Russian	Non-English language		
92	29719871	2018	Clinical study	Other studies didn't meet criteria		
93	29350768	2018	Clinical study	Other studies didn't meet criteria		
94	26549775	2016	Clinical study	Other studies didn't meet criteria		
95	28125762	2017	Meta-analysis	Not-original research		
96	27102333	2016	Clinical study	Other studies didn't meet criteria		
97	30735238	2019	Clinical study	Other studies didn't meet criteria		
98	29506183	2018	Clinical study	Other studies didn't meet criteria		
99	29482339	2018	Clinical study	Other studies didn't meet criteria		
100	31373365	2019	Review 18	Not-original research		
101	30538180	2018	Clinical study	Other studies didn't meet criteria		
102	29653862	2019	Clinical study	Other studies didn't meet criteria		
103	27151248	2016	Clinical study	Other studies didn't meet criteria		
104	31253623	2019	Clinical study	Other studies didn't meet criteria		
105	26511097	2016	Clinical study	Other studies didn't meet criteria		
106	30399404	2019	Clinical study	Other studies didn't meet criteria		
107	29109959	2017	Review 19	Not-original research		
108	28531784	2016	Clinical study	Other studies didn't meet criteria		
109	29373513	2018	Selected article	Cohort studies included	Figure 5: Study f	
110	26460205	2016	Clinical study	Other studies didn't meet criteria		
111	27510655	2017	Clinical study	Other studies didn't meet criteria		
112	25418803	2015	Fundamental research	Non-clinical studies		
113	31140389	2019	Review 20	Not-original research		
114	30404694	2018	Chinese	Non-English language		
115	29852087	2018	Review 21	Not-original research		
116	30355801	2018	Clinical study	Other studies didn't meet criteria		
117	30984765	2019	Review 22	Not-original research		
118	31965846	2019	Clinical study	Other studies didn't meet criteria		
119	29989465	2019	Clinical study	Other studies didn't meet criteria		
120	30773108	2019	Review 23	Not-original research		
121	28931089	2017	Clinical study	Other studies didn't meet criteria		
122	30471188	2019	Clinical study	Other studies didn't meet criteria		
123	29571566	2019	Clinical study	Other studies didn't meet criteria		
124	30901505	2019	Clinical study	Other studies didn't meet criteria		
125	30400238	2018	Clinical study	Other studies didn't meet criteria		
126	31187868	2019	Clinical study	Other studies didn't meet criteria		
127	30269035	2018	Selected article	Cohort studies included	Figure 5: Study d	
128	31551328	2019	Clinical study	Other studies didn't meet criteria		
129	31238287	2019	Clinical study	Other studies didn't meet criteria		
130	23898195	2013	Fundamental research	Non-clinical studies		
131	31377126	2019	Clinical study	Other studies didn't meet criteria		
132	28814395	2017	Clinical study	Other studies didn't meet criteria		
133	30638420	2019	Clinical study	Other studies didn't meet criteria		
134	31776537	2019	Selected article	Cohort studies included	Figure 5: Study p	

135	29893876	2018	Fundamental research	Non-clinical studies		
136	23038174	2013	Selected article	Cohort studies included	Figure 5: Study g	
137	31172651	2019	A case report	Non-clinical studies		
138	29936607	2018	Clinical study	Other studies didn't meet criteria		
139	31666478	2019	Clinical study	Other studies didn't meet criteria		
140	30264073	2018	Clinical study	Other studies didn't meet criteria		
141	31615057	2019	Clinical study	Other studies didn't meet criteria		
142	31710785	2019	Russian	Non-English language		
143	32024556	2020	Review 24	Not-original research		
144	31517324	2019	Clinical study	Other studies didn't meet criteria		
145	29973712	2018	Clinical study	Other studies didn't meet criteria		
146	30394894	2019	Review 25	Not-original research		
147	30747307	2019	Review 26	Not-original research		
148	28228424	2017	Clinical study	Other studies didn't meet criteria		
149	26578751	2015	Review 27	Not-original research		
150	30358831	2018	Clinical study	Other studies didn't meet criteria		
151	30541093	2018	Clinical study	Other studies didn't meet criteria		
152	27974055	2016	Clinical study	Other studies didn't meet criteria		
153	31373737	2019	Fundamental research	Non-clinical studies		
154	30803510	2019	Clinical study	Other studies didn't meet criteria		
155	31412673	2019	Review 28	Not-original research		
156	26599039	2016	Clinical study	Other studies didn't meet criteria		
157	28285654	2017	Clinical study	Other studies didn't meet criteria		
158	28744326	2017	Clinical study	Other studies didn't meet criteria		
159	30948911	2019	Clinical study	Other studies didn't meet criteria		
160	30867067	2019	Clinical study	Other studies didn't meet criteria		
161	31271261	2019	Clinical study	Other studies didn't meet criteria		
162	28230784	2017	Clinical study	Other studies didn't meet criteria		
163	31634399	2019	Fundamental research	Non-clinical studies		
164	31370734	2019	Clinical study	Other studies didn't meet criteria		
165	31829172	2019	Introduction of study protocol	Other studies didn't meet criteria		
166	27810310	2016	Clinical study	Other studies didn't meet criteria		
167	28940737	2017	Clinical study	Other studies didn't meet criteria		
168	30396006	2019	Clinical study	Other studies didn't meet criteria		
169	29558912	2018	Fundamental research	Non-clinical studies		
170	30939160	2019	Clinical study	Other studies didn't meet criteria		
171	26249791	2016	Clinical study	Other studies didn't meet criteria		
172	30423561	2019	Clinical study	Other studies didn't meet criteria		
173	28624933	2018	Clinical study	Other studies didn't meet criteria		
174	29452584	2018	Clinical study	Other studies didn't meet criteria		
175	27782071	2016	Clinical study	Other studies didn't meet criteria		
176	32002758	2020	Review 29	Not-original research		
177	26455903	2015	Clinical study	Other studies didn't meet criteria		
178	31547446	2019	Clinical study	Other studies didn't meet criteria		
179	31841548	2019	Clinical study	Other studies didn't meet criteria		
180	27018166	2016	Clinical study	Other studies didn't meet criteria		

181	27606833	2017	Clinical study	Other studies didn't meet criteria		
182	27824805	2016	Review 30	Not-original research		
183	29509315	2019	Clinical study	Other studies didn't meet criteria		
184	29493330	2018	Review 31	Not-original research		
185	29624599	2018	Clinical study	Other studies didn't meet criteria		
186	30527258	2018	Clinical study	Other studies didn't meet criteria		
187	27966574	2017	Clinical study	Other studies didn't meet criteria		
188	30056386	2018	Clinical study	Other studies didn't meet criteria		
189	30139999	2018	Clinical study	Other studies didn't meet criteria		
190	28390574	2017	Review 32	Not-original research		
191	25519526	2015	Clinical study	Other studies didn't meet criteria		
192	28558792	2017	Fundamental research	Non-clinical studies		
193	29114246	2017	Clinical study	Other studies didn't meet criteria		
194	28591831	2017	Clinical study	Other studies didn't meet criteria		
195	29378051	2018	Clinical study	Other studies didn't meet criteria		
196	27502158	2016	Clinical study	Other studies didn't meet criteria		
197	29274690	2018	Clinical study	Other studies didn't meet criteria		
198	29873593	2018	Clinical study	Other studies didn't meet criteria		
199	29453751	2019	Clinical study	Other studies didn't meet criteria		
200	27676396	2016	Clinical study	Other studies didn't meet criteria		
201	30003647	2018	Clinical study	Other studies didn't meet criteria		
202	30052094	2018	Review 33	Not-original research		
203	25690418	2015	Selected article	Cohort studies included	Figure 5: Study h	
204	30502656	2019	Clinical study	Other studies didn't meet criteria		
205	30665298	2019	Fundamental research	Non-clinical studies		
206	30845997	2019	Selected article	Cohort studies included	Figure 5: Study o	
207	32199523	2020	Clinical study	Other studies didn't meet criteria		
208	32211860	2020	Systematic review 34	Not-original research		
209	27572508	2016	Clinical study	Other studies didn't meet criteria		
210	28069076	2016	Clinical study	Other studies didn't meet criteria		
211	30826445	2019	A case report	Non-clinical studies		
212	27708392	2016	Clinical study	Other studies didn't meet criteria		
213	28873671	2017	Fundamental research	Non-clinical studies		
214	23594389	2013	Review 35	Not-original research		
215	27504897	2016	Clinical study	Other studies didn't meet criteria		
216	27744545	2018	Translational research	Non-clinical studies		
217	28639601	2017	Clinical study	Other studies didn't meet criteria		
218	29464347	2019	Clinical study	Other studies didn't meet criteria		
219	29511074	2018	Fundamental research	Non-clinical studies		
220	32174888	2020	Review 36	Not-original research		
221	28677210	2017	Clinical study	Other studies didn't meet criteria		
222	26607554	2015	Review 37	Not-original research		
223	27088328	2016	Review 38	Not-original research		
224	29332901	2017	Clinical study	Other studies didn't meet criteria		
225	30006229	2018	Fundamental research	Non-clinical studies		
226	28937980	2017	Clinical study	Other studies didn't meet criteria		

227	28555008	2017	Clinical study	Other studies didn't meet criteria		
228	24416266	2014	Analysis based on public database	Not-original research		
229	32155205	2020	Fundamental research	Non-clinical studies		
230	27518814	2016	Fundamental research	Non-clinical studies		
231	31851298	2020	Clinical study	Other studies didn't meet criteria		
232	26272781	2015	Clinical study	Other studies didn't meet criteria		
233	29198188	2017	Clinical study	Other studies didn't meet criteria		
234	29490103	2018	Clinical study	Other studies didn't meet criteria		
235	28971851	2017	Clinical study	Other studies didn't meet criteria		
236	28296348	2017	Selected article	Cohort studies included	Figure 5: Study i	
237	24233256	2015	Clinical study	Other studies didn't meet criteria		
238	23075436	2012	Fundamental research	Non-clinical studies		
239	26265295	2015	Clinical study	Other studies didn't meet criteria		
240	25484891	2014	Review 39	Not-original research		
241	28954842	2017	Clinical study	Other studies didn't meet criteria		
242	25258407	2014	Clinical study	Other studies didn't meet criteria		
243	30669671	2019	Clinical study	Other studies didn't meet criteria		
244	27585552	2016	Translational research	Non-clinical studies		
245	24690120	2014	Clinical study	Other studies didn't meet criteria		
246	28553274	2017	Clinical study	Other studies didn't meet criteria		
247	28164731	2017	Selected article	Cohort studies included	Figure 5: Study k	
248	26228065	2015	Clinical study	Other studies didn't meet criteria		
249	25714718	2015	Environmental research	Non-clinical studies		
250	27633737	2016	Clinical study	Other studies didn't meet criteria		
251	31971861	2020	Clinical study	Other studies didn't meet criteria		
252	22649263	2012	Selected article	Cohort studies included	Figure 5: Study l	
253	26235304	2015	Clinical study	Other studies didn't meet criteria		
254	25903259	2016	Clinical study	Other studies didn't meet criteria		
255	30664020	2019	Clinical study	Other studies didn't meet criteria		
256	26859894	2016	Socioeconomic research	Non-clinical studies		
257	28157671	2016	Introduction of study protocol	Other studies didn't meet criteria		
258	31996717	2020	Clinical study	Other studies didn't meet criteria		
259	26919743	2016	Clinical study	Other studies didn't meet criteria		
260	28418214	2017	Clinical study	Other studies didn't meet criteria		
261	27500753	2016	Clinical study	Other studies didn't meet criteria		
262	28821315	2017	Clinical study	Other studies didn't meet criteria		
263	32028108	2020	Clinical study	Other studies didn't meet criteria		
264	25332326	2014	Clinical study	Other studies didn't meet criteria		
265	28012242	2017	Clinical study	Other studies didn't meet criteria		
266	26426642	2015	Clinical study	Other studies didn't meet criteria		
267	28902124	2017	Clinical study	Other studies didn't meet criteria		
268	26940357	2017	Clinical study	Other studies didn't meet criteria		
269	24995004	2014	Fundamental research	Non-clinical studies		
270	20962874	2011	Fundamental research	Non-clinical studies		
271	25299601	2014	Selected article	Cohort studies included	Figure 5: Study j	

272	25435420	2015	Clinical study	Other studies didn't meet criteria		
273	28655159	2017	Clinical study	Other studies didn't meet criteria		
274	31761826	2020	Fundamental research	Non-clinical studies		
275	26987626	2016	Clinical study	Other studies didn't meet criteria		
276	25438151	2014	Clinical study	Other studies didn't meet criteria		
277	26862979	2016	Translational research	Non-clinical studies		
278	20339542	2010	Fundamental research	Non-clinical studies		
279	27038949	2016	Fundamental research	Non-clinical studies		
